# Potential synaptic plasticity‐based Shenzhiling oral liquid for a SAD Mouse Model

**DOI:** 10.1002/brb3.1385

**Published:** 2019-08-20

**Authors:** Yahan Wang, Pengwen Wang, Fang Chen, Mana Lulu, Shuaiyang Huang, Zhenhong Liu

**Affiliations:** ^1^ Key Laboratory of Chinese Internal Medicine of Ministry of Education and Beijing Dongzhimen Hospital Beijing University of Chinese Medicine (BUCM) Beijing China; ^2^ Key Laboratory of Pharmacology of Dongzhimen Hospital (BUCM) State Administration of Traditional Chinese Medicine Beijing China; ^3^ Rehabilitation centre The Xinjiang Uygur Autonomous Region Traditional Chinese Medicine Hospital affiliated to Xinjiang Medical University Urumqi China

**Keywords:** glutamate receptors, GSK3β, Shenzhiling oral liquid, sporadic Alzheimer's disease, synapse

## Abstract

**Background:**

Synaptic plasticity is the basis of memory formation. The pathological manifestations of abnormal glucose metabolism in the nervous system of sporadic Alzheimer's disease (SAD) may affect synaptic plasticity, thus causing memory damage. As a traditional Chinese medicine compound preparation, the mechanism by which Shenzhiling (SZL) oral liquid can alleviate the cognitive impairment of SAD by improving synaptic plasticity remains unclear. Objective: This article mainly discusses whether SZL can exert a protective synaptic effect as mediated by glutamate receptors and glycogen synthesis kinase 3β (GSK3β); further, it discusses whether SZL can improve cognitive function in SAD.

**Methods:**

C57BL/6 mice were used as a SAD model after injection with streptozotocin (STZ) into the bilateral lateral ventricles; mice of the same background were injected with artificial cerebrospinal fluid into bilateral ventricles and were used as a control group. After 3 months of exposure to the intervention, the step‐down test was carried out. Western blot was used to detect the levels of NMDAR2B, p‐NMDAR2B, mGlu5, GSK3β, and p‐GSK3β in the hippocampus of mice. Immunohistochemical analysis was used to observe the number of GSK3β‐positive cells in the CA1 region of mouse hippocampus.

**Results:**

The memory retention ability of mice was significantly improved after 3 months of SZL treatment, and the expression levels of NMDAR2B, mGlu5, and GSK3β were significantly changed.

**Conclusion:**

Shenzhiling provides a potential for the treatment for SAD with traditional Chinese medicine.

## 1 INTRODUCTION

The degeneration of memory and learning ability is the main pathological manifestation of early‐stage Alzheimer's disease (AD). Memory formation begins at the synaptic nerve membrane. In excitatory synapses, the stimulation of presynaptic neurons causes the release of glutamate, which activates both ionic glutamate receptors (iGluRs) and metabotropic glutamate receptors (mGluRs) located on the postsynaptic spine. Subsequently, large amounts of Ca^2+^ flood into the postsynaptic neurons. This process induces long‐term potentiation (LTP), an important mechanism for memory formation at the synaptic level. Insulin receptors (IRs) are abundant in the central nervous system (CNS), especially in hippocampal synapses. Insulin signals may affect synaptic plasticity by regulating the expression and transport of glutamate receptors. Studies have shown that the deficiency of insulin receptor substrate 2 (IRS‐2) leads to dysfunction of N‐methyl‐D‐aspartic acid (NMDA) receptors after LTP induction. NMDA receptors, members of the iGluR family, are glutamate‐gated ion channels found throughout the brain that play an important role in synaptic plasticity (Martín et al., 2012).

Recent studies have shown that dysfunctional insulin signals contribute to the pathogenesis of AD (Craft, 2007; Monte & Wands, 2005). Most sporadic Alzheimer's disease (SAD) insulin‐signaling disorders in the brain are at the heart of the neurodegenerative cascade. The understanding of the pathogenesis of AD with impaired insulin signaling in memory contributes to the view of AD as a neuroendocrine disease. Insulin resistance (IR) and insulin deficiency in the brain play a key role in the cognitive dysfunction of AD, and some scholars even believe that AD is type 3 diabetes mellitus (T3DM; Monte & Wands, 2008; Steen et al., 2005).

To better understand the pathological mechanism of abnormal glucose metabolism in the CNS in the context of SAD, Sharma et al. injected streptozotocin (STZ) into the lateral ventricle of mice, which resulted in an obvious decline in learning and memory ability in addition to disordered brain metabolism. This approach has become a commonly used sporadic AD research model (Sharma & Gupta, 2001). STZ is a nitrosourea compound, and bilateral lateral ventricle injection generates a slow decrease in glucose utilization and glycogen metabolism in cerebral cortex and hippocampus, causing a decline in the oxidative metabolism of the CNS and suppression of insulin receptors, eventually leading to the progressive deterioration of cognitive function.

Shenzhiling (SZL) oral liquid is the first Chinese medicine compound approved as a new drug for ADby the State Food and Drug Administration (SFDA), filling a gap for a compound natural AD drug at home and abroad, but the mechanism by which it treats mild‐to‐moderate dementia remains unclear. In this study, donepezil was selected as the positive control drug; this drug may function by reversibly inhibiting the hydrolysis of acetylcholine by cholinergic nerves by enhancing nerve function, resulting in an increase in the concentration of acetylcholine. Acetylcholine acts on the postsynaptic membranes and is closely related to learning and memory.

## 2 MATERIALS

### 2.1 Animals

A total of 105 male 3‐month‐old C57BL/6 wild‐type mice were purchased from Beijing Vital River Laboratory Animal Technology Co., Ltd., license [SCXK (Beijing) 2012‐0001], weighing 22–25 g. All mice were kept in an isolated animal room of the Traditional Chinese Medicine Pharmacology Laboratory of Dongzhimen Hospital, license [SXXK (Beijing) 2015‐0001]. The mice were housed individually at a temperature of 20–22°C, relative humidity of 60%−70%, and a 12:12 hr light–dark cycle (light time 7:00–19:00).

### 2.2 Model and groups

After 12 hr of fasting, the animals were subjected to intraperitoneal anesthesia with 4% chloral hydrate (0.1 ml/10 g), and the anesthetized mice were fixed on the stereoscopic locator. The skull was drilled with a cone cranium to expose the dura at 1.5 and 1.0 mm behind the anterior fontanel and 1.5 mm from the sagittal suture. Injections of 5 µl of liquid were made over a period of 5 min using a needle lowered vertically to 2 mm below the brain surface. The needle was subsequently withdrawn, and the incision was sutured. In the control group, artificial cerebrospinal fluid (volume equal to STZ solution) was injected bilaterally into the lateral ventricles on day 1 and day 3, and another 90 mice were injected into the lateral ventricles on day 1 and day 3 with streptozotocin (STZ) solution (prepared with artificial cerebrospinal fluid, concentration 6 mg/ml, injection volume 3 mg/kg). After mold making, antibiotics were given for three consecutive days, and after adaptive feeding for one week, a step‐down test was carried out to eliminate the failed models.

In addition to the CMC‐treated control group, the mice were randomly divided into the following five groups and were treated continuously for three months: donepezil‐treated group (donepezil 0.92 mg kg^−1^ day^−1^ administered orally), SZL oral liquid high‐dose treatment group (SZL 300 mg kg^−1^ day^−1^ administered orally), SZL oral liquid medium‐dose treatment group (SZL 150 mg kg^−1^ day^−1^ administered orally) and SZL oral liquid low‐dose treatment group (SZL 75 mg kg^−1^ day^−1^ administered orally), and CMC model group (0.5% CMC of the same volume administered orally). The CMC control group was fed the same as the model group.

### 2.3 Drug treatment

For the control and model groups, 0.5% sodium carboxymethyl cellulose (CMC) was prepared with distilled water. SZL oral liquid was purchased from Shandong Wohua Pharmaceutical Co. Ltd. (batch number: Z20120010) and dissolved in 0.5% CMC at doses of 1.3, 2.6, and 5.2 mg kg^−1^ day^−1^. Donepezil was purchased from Eisai Pharmaceutical Co. Ltd (batch number 140635) and dissolved in 0.5% CMC at a dose of 0.92 mg kg^−1^ day^−1^. Mice were continuously administered the solutions at a volume of 0.1 ml/10 g/day for 3 months. After the 3‐month intervention and behavioral tests, all the mice were sacrificed, and their tissues were used for further biochemical and immunohistochemical tests.

### 2.4 Antibodies and chemicals

The primary antibodies used were as follows: Rabbit monoclonal anti‐Metabotropic Glutamate Receptor 5 antibody (abcam, 1:3,000 dilution for WB, 1:500 dilution for IHC‐P), NMDAR2B rabbit monoclonal antibody (Abcam, 1:1,000 dilution for WB, 1:1,000 dilution for IHC‐P), p‐NMDAR2B rabbit monoclonal antibody (Abcam, 1:1,000 dilution for WB), GSK3β rabbit monoclonal antibody (Cell Signaling, 1:1,000 dilution for WB), and p‐GSK3β rabbit monoclonal antibody (Cell Signaling, 1:1,000 dilution for WB, 1:100 dilution for IHC‐P).

The SABC immunohistochemical staining kit, DAB color kit (Wuhan, BOSTER Biological Technology Co. Ltd.), general Western blot reagents, SDS‐PAGE gel preparation kit, RIPA cell lysis buffer, Tris‐base, sodium lauryl sulfate (SDS), sulfuric acid amine (ammonium persulfate, APS), powdered skim milk, and glycine were bought from Beijing Huanyataike Biomedical Technology Co. Ltd. The ECL supersensitive substrate chemiluminescence detection kit was purchased from the Sunbio Biomedical Technology Co. Ltd. PVDF membranes were purchased from Millipore (USA).

## 3 METHOD

### 3.1 Behavioral test

The step‐down test was used to assess memory in mice. This experiment was divided into training and trial period over two days, recording the time required to jump off the platform for the first time (step‐down latency, SDL) and the number of times jumping off the platform (error time, ET) in the trial period. If the mouse did not jump off the platform, ET was recorded as 0, and SDL was the observation time.

### 3.2 Fresh tissue specimens

After sacrifice, hippocampal tissue was dissected on ice. The hippocampal tissue was then placed in EP tubes and stored in a liquid nitrogen tank at −180°C. Each group had nine mice, and hippocampal tissue was collected from both hemispheres.

### 3.3 Western blotting

After putting the protein face of the sample up and placing it on the clean plastic film, the experimental procedure including protein extraction, protein electrophoresis, membrane transfer, hybridization, and colorimetric detection was conducted. First, the preconfigured light‐emitting detection fluid was carefully transferred to the protein membrane, ensuring that the fluid covered the protein membrane uniformly. Then, the protein membrane was incubated for 1 ~ 2 min at room temperature while ensuring no bubbles formed. Then, the protein membrane was placed in the X‐ray film cassette (this process was conducted in a dark room with some light was allowed). In a closed dark room, the X‐ray film was placed onto the packed membrane; the film cassette was closed, and the film was exposed for 30 s to 1 min. The film was developed, and the exposure time was shortened or lengthened on the next X‐ray film according to its exposure intensity. Then, the films were scanned and stored as TIF images using the HP Scanjet G 4050 scanner. Finally, the results were analyzed by Quantity One software. The final result was obtained by analyzing the bands of the different groups and comparing the integrated grayscale of these bands with that of internal reference (β‐actin), using the β‐actin ID date to unify the data of different groups to obtain the result in the form of percentage (i.e., ID/internal reference ID x 100%).

### 3.4 Paraffin section

The mice were sacrificed after the behavior test and perfused with polyformaldehyde. All fixed brains were dehydrated, paraffin embedded, and sliced. The CA1 region of each hippocampus was sequentially sliced. The thickest region of each slice was 4 μm, and there were six mice in each group.

### 3.5 Immunohistochemical detection and analysis

Six brain slices were taken from each group and observed three times under a light microscope to calculate the number of positive neurons in the hippocampal CA1 region. The Image‐Pro Plus image analysis software was used to record the number of positive cells in each group.

### 3.6 Statistical analysis

SPSS 19.0 was used for statistical analysis of the data. Single factor ANOVA was performed followed by a post hoc multiple comparison test. Since no heterogeneity of variance was observed in any of the parameters tested, the least significant difference (LSD) test was used to examine the differences between the groups. *p* < .05 was considered statistically significant.

## 4 RESULTS

### 4.1 STZ can lead to abnormal synaptic structure in hippocampus

**Figure 1:**
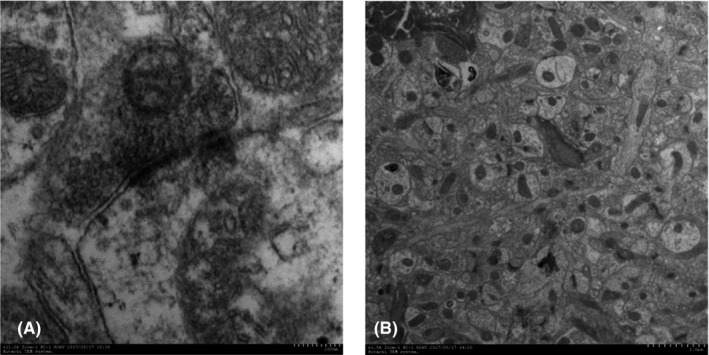
Pathological changes of synaptic structure in model group. The structure of presynaptic membrane and synaptic gap is not clear. The morphology and structure of dense plaques attached on the cytoplasmic surface of the postsynaptic membrane are abnormal, showing irregular thickening, or poor continuity and incomplete (A). The number of synapses in hippocampus decreased (B)

We observed the changes in synapses in hippocampus of mice under transmission electron microscope. Clear synaptic vesicles were dense and disordered in the hippocampal CA1 area of the model group. The structures of the presynaptic membranes and synaptic gaps were not clear. The morphological structure of dense plaques attached to the postsynaptic membrane cytoplasm was abnormal, showing irregular thickening or poor continuity and incompleteness (Figure 1A). The number of synapses in the hippocampal nerve blanket was decreased, with variation seen in the sizes of synapses (Figure 1B).

### 4.2 SZL improves memory retention in SAD mice

**Figure 2:**
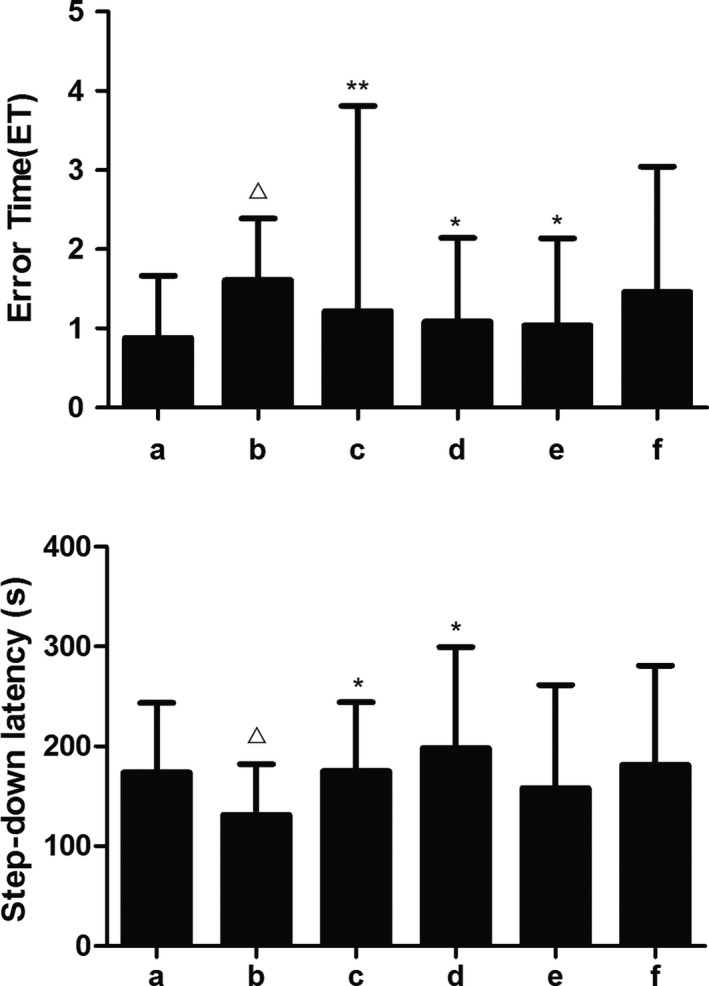
The performance of each group in the step‐down test. a, control group; b, model group; c, donepezil group; d, SZL high‐dose group; e, SZL medium‐dose group; f, SZL low‐dose group. The ET decreased and the SDL increased after donepezil and SZL high‐dose treatment. Compared with control group, ^△^ *p* ^^< .05; compared with model group, *p < .05, **p < .01

During the trial period of the step‐down test, the step‐down latency of the model group was significantly shortened, and the number of ET was significantly increased. After receiving donepezil and SZL treatments, the SDL was significantly increased, and the number of ET was decreased; this result indicated that high‐dose and medium‐dose SZL treatment can improve the ability of the mice to avoid electric stimulation, thus improving memory formation, with an effectiveness comparable to donepezil (Figure 2).

### 4.3 SZL oral liquid promoted synaptogenesis

**Figure 3:**
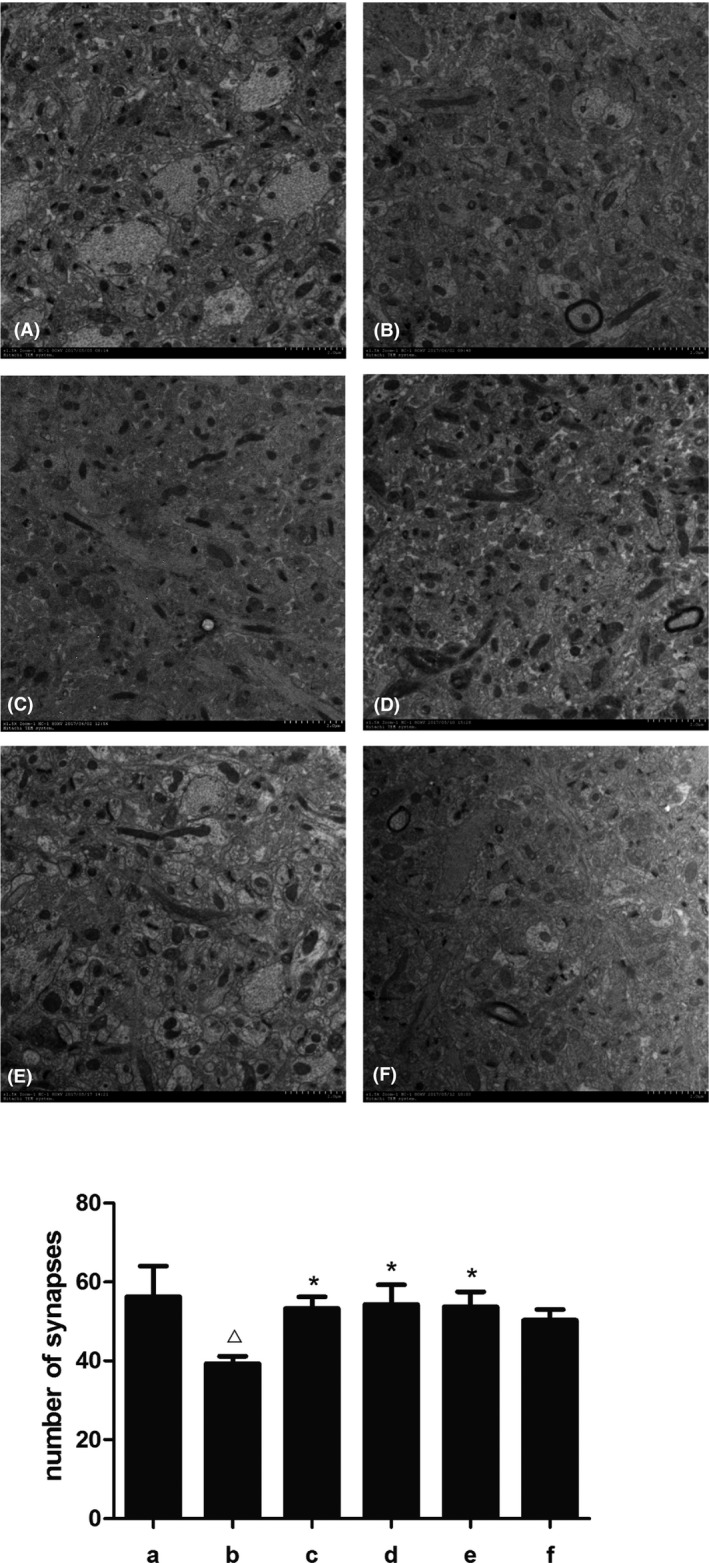
A: (A)‐(F) The number of synapses in each group was observed under transmission electron microscope. B:The number of synapses in hippocampus of each group. a, control group; b, model group; c, donepezil group; d, SZL high‐dose group; e, SZL medium‐dose group; f, SZL low‐dose group. The number of synapses in hippocampus decreased in model group, while in the donepezil, high‐dose and mediumdose SZL group it increased. Compared with control group, ^△^ *p * < .05; compared with model group, *p < .05

We further analyzed the number of synapses in the hippocampus of each group. We found that the number of synapses in the untreated model group decreased significantly. After donepezil treatment, the number of synapses in the hippocampal regions increased significantly. Among the SZL‐treated mice, the number of synapses was increased in the high‐dose group and the medium‐dose group, but no significant increase was observed in the low‐dose group (Figure 3).

### 4.4 SZL oral liquid increased synaptic plasticity

**Figure 4:**
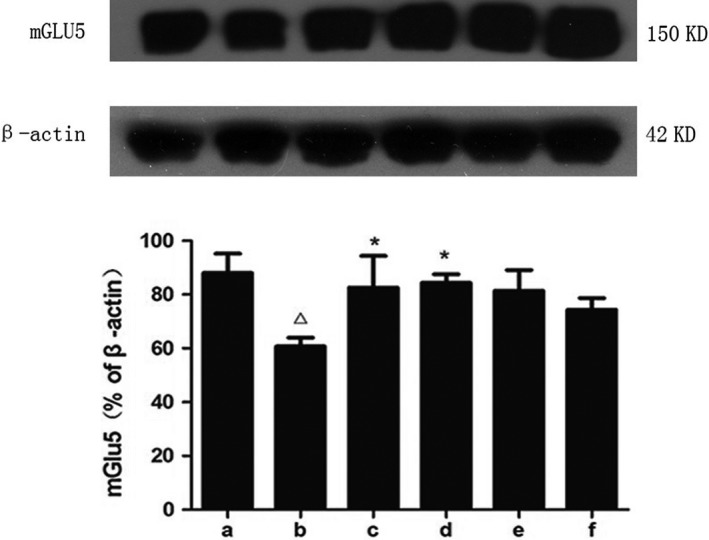
Expression of mGlu5 in each group. a, control group; b, model group; c, donepezil group; d, SZL high‐dose group; e, SZL medium‐dose group; f, SZL low‐dose group. The expression of mGlu5 decreased after STZ injection, and the donepezil and high‐dose SZL treatment increased it. Compared with control group, ^△^ *p *< .05; compared with model group, *p <.05

Glutamate is an important excitatory amino acid neurotransmitter in the CNS and plays an important role in synapse formation and LTP (Pascual, Ben Achour, Rostaing, Triller, & Bessis, 2012). Metabotropic glutamate receptor 5 (mGlu5) is an exciting research target that promises to improve cognition. Studies have found that the LTP induced by activation of the M1 acetylcholine receptor depends on the synaptic activation of mGlu5 during its activation (Ghoshal et al., 2017). Western blotting was used to detect the expression level of mGlu5 (Figure 4). The level of mGlu5 was significantly reduced in the untreated model group, and it was increased in all groups after drug treatment, especially in the SZL high‐dose group.

**Figure 5:**
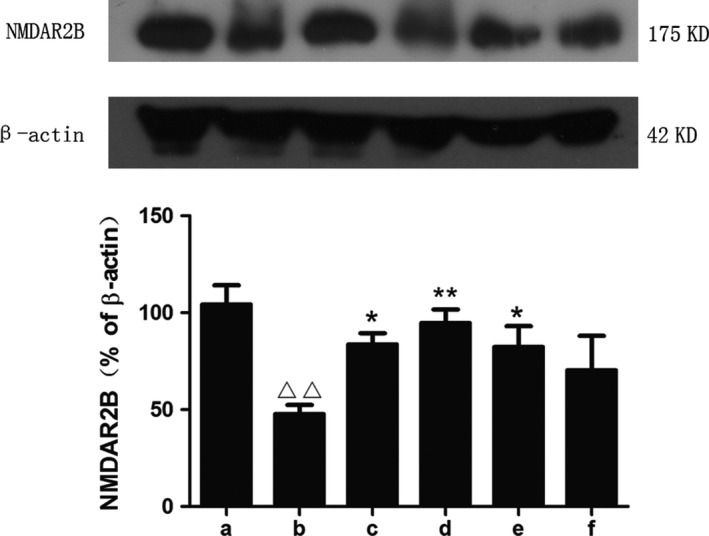
Expression of NMDAR2B in each group. a, control group; b, model group; c, donepezil group; d, SZL high‐dose group; e, SZL medium‐dose group; f, SZL low‐dose group. The expression of NMDAR2B decreased after STZ injection. Besides the donepezil and medium‐dose SZL treatment increased the expression of NMDAR2B, the high‐dose SZL treatment increased it most significantly. Compared with control group, ^△△^ *p* < .01; compared with model group, *p < .05, **p < .01

**Figure 6:**
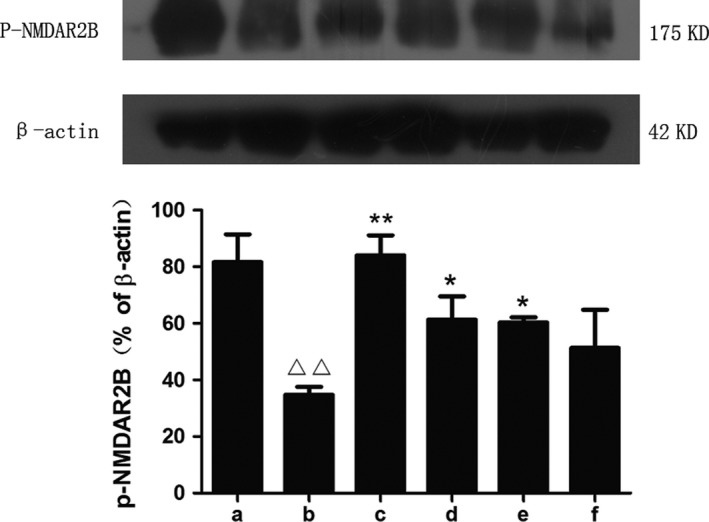
Expression of p‐NMDAR2B in each group. a, control group; b, model group; c, donepezil group; d, SZL high‐dose group; e, SZL medium‐dose group; f, SZL low‐dose group. The expression of p‐NMDAR2B decreased after STZ injection. After the treatment of donepezil, high‐dose and medium‐dose SZL, the expression of p‐NMDAR2B increased, among which the donepezil group increased most significantly. Compared with control group, ^△△^ *p* < .01; compared with model group, *p < .05, **p < .01

mGlu5 has a close partnership with the NMDARs in terms of regulating synaptic plasticity (Zuena et al., 2018).We found that, with the influence of STZ on the mGlu5 level of the model group, the expression of p‐NMDA2B presented the same downward trend, and the expression of NMDAR2B increased. Subsequently, SZL and donepezil also increased the expression of p‐NMDAR2B and decreased the NMDAR2B level in the drug treatment groups, among which the changes were most obvious in the SZL high‐dose group, while this effect was not obvious in the SZL low‐dose group (Figures 5 and 6).

Recent studies have shown that in addition to NMDAR2B and mGlu5, GSK3β can be involved in synaptic plasticity by influencing LTP and LTD. GSK3β is inhibited during LTP induction (Hooper et al., 2007; Peineau et al., 2007). In transgenic mice overexpressing GSK3β, LTP is inhibited, which affects the memory formation ability of mice (Hernandez, Borrell, Guaza, Avila, & Lucas, 2002).

**Figure 7:**
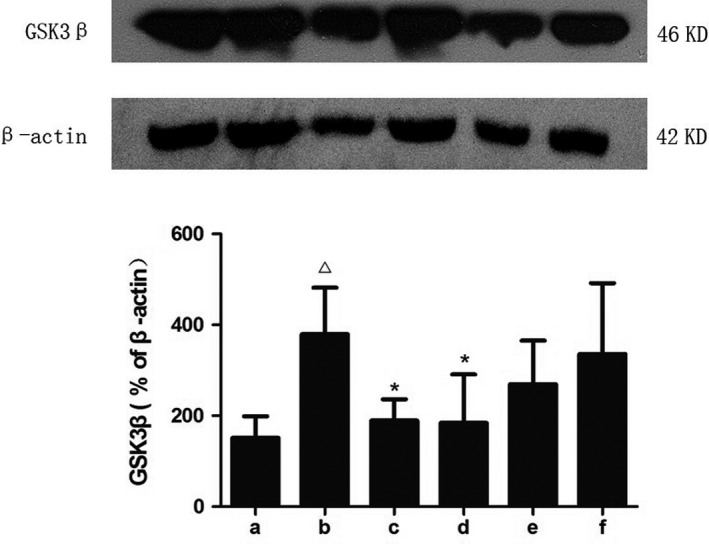
Expression of GSK3β in each group. a, control group; b, model group; c, donepezil group; d, SZL high‐dose group; e, SZL medium‐dose group; f, SZL low‐dose group. The expression of GSK3β increased after STZ injection and the donepezil and high‐dose SZL treatment decreased it. Compared with control group, ^△^ *p* < .05; compared with model group, *p ^^< .05

**Figure 8:**
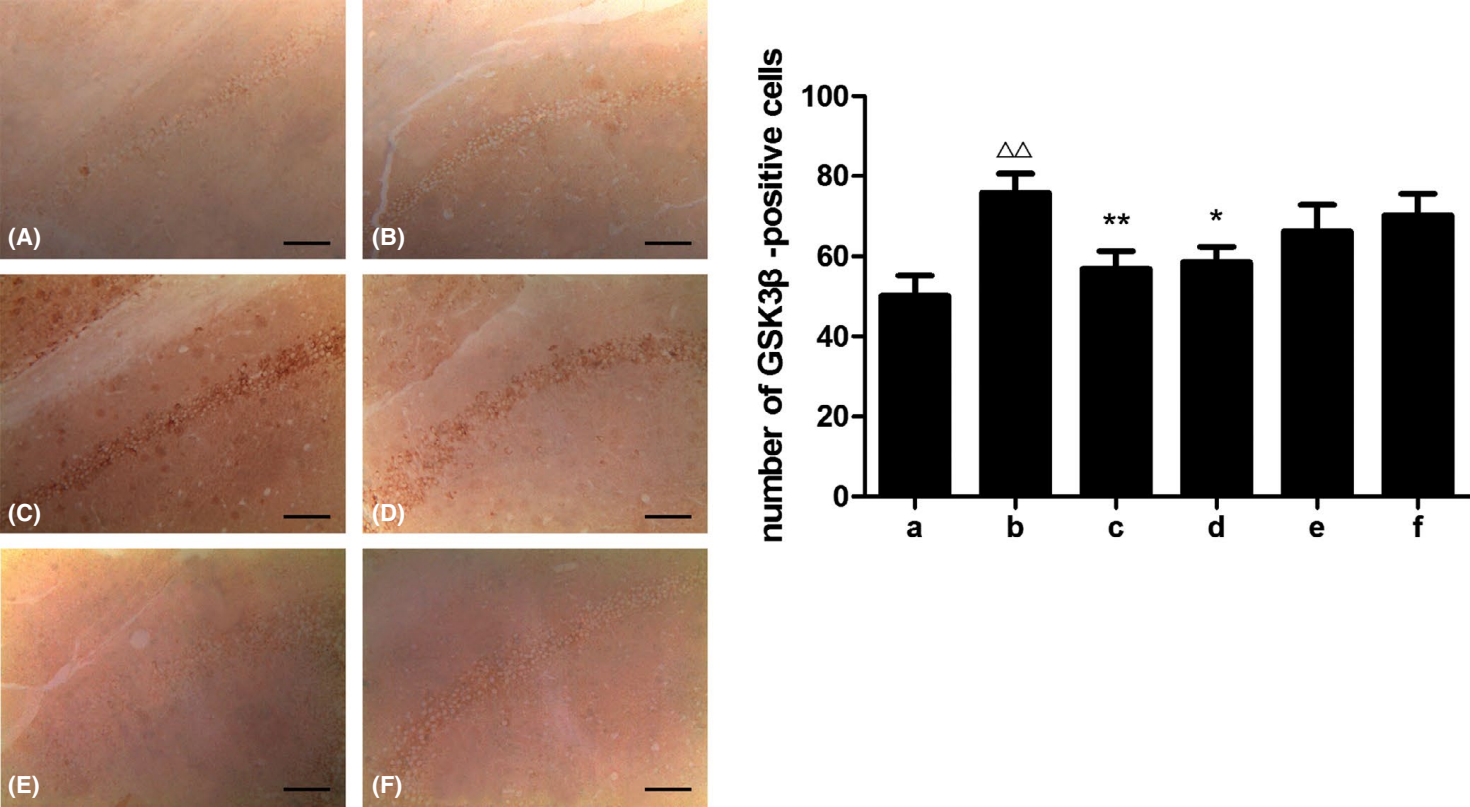
A: (A)‐(F) Immunohistochemical staining of hippocampus GSK3β. B:Number of GSK3β‐positive cells in each group. a, control group; b, model group; c, donepezil group; d, SZL high‐dose group; e, SZL medium‐dose group; f, SZL low‐dose group. Consistent with the WB result of GSK3β, its expression increased after STZ injection and the donepezil and high‐dose SZL treatment decreased it. Compared with control group, ^△△^ *p* < .01; compared with model group, *p < .05, **p < .01

**Figure 9:**
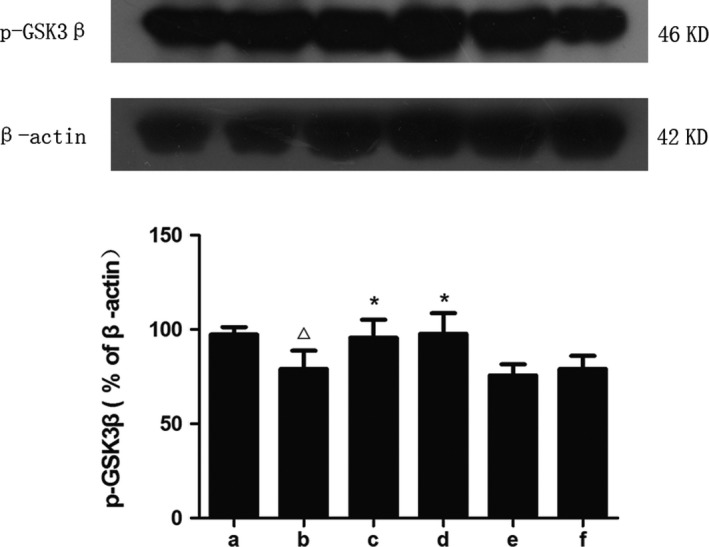
Expression of p‐GSK3β in each group. a, control group; b, model group; c, donepezil group; d, SZL high‐dose group; e, SZL medium‐dose group; f, SZL low‐dose group. The expression of p‐GSK3β decreased after STZ injection and the donepezil and high‐dose SZL treatment increased it. Compared with control group, ^△^ *p *< .05; compared with model group, *p < .05

In this study, we assessed the levels of GSK3β and p‐GSK3β (Figures 7, 8, 9). STZ bilateral intraventricular injection resulted in an increase in GSK3β expression and a decrease in p‐GSK3β expression in mice. A significant decrease in GSK3β expression and a significant increase in p‐GSK3β expression were observed in the hippocampus after treatment with donepezil or high‐dose SZL, which was consistent with the abovementioned theory.

## 5 DISCUSSION

Shenzhiling bilateral ventricular injection can successfully simulate the pathological manifestations of insulin resistance in the central nervous system (CNS), which is an important pathological feature of SAD, especially in patients with early cognitive impairment (Grieb, 2015). Insulin receptors (IRs) are widespread in the CNS, especially in the hippocampus. Cognitive impairment associated with insulin resistance (IR) was observed in both T2DM and AD animal models. The correlation between abnormal central glucose metabolism and cognitive decline was also observed clinically and epidemiologically (Freiherr et al., 2013; Li, Aou, Hori, & Oomura, 2002; Vandal et al., 2014; Winocur et al., 2005). Memory and information acquisition are stored at synapses in the form of changes in synaptic efficiency. Synaptic plasticity plays an important role in memory and learning ability, especially in long‐term potentiation (LTP) and long‐term depression (LTD), two forms of synaptic plasticity. Studies have found that a decrease in insulin receptors in the hippocampus leads to a decrease in synaptic plasticity (Grillo et al., 2015).Consistently, in our study, pathological changes and decreased numbers of synapses were observed in the synaptic ultrastructure of the model group; at the same time, the commonly used step‐down test was selected to evaluate the effects of drugs on learning and memory of mice. Their memory preservation ability was significantly decreased in the behavioral test, which confirmed that STZ could damage the synaptic structure and function and cause related cognitive impairment.

Aspartic acid and glutamate are Ca^2+^‐dependent excitatory neurotransmitters in mammalian brain, which play a role after binding to glutamate receptors. Glutamate receptors can be divided into the iGluRs and mGluRs. The iGluRs include the N‐methyl‐D‐aspartic acid (NMDA) receptor, the α‐ amino‐3‐carboxyl‐5‐methyl‐isooxazole‐4‐yl propanoic acid (AMPA) receptor and the kainate (KA) receptor, while the mGluRs include eight subtypes. NMDARs are distributed in both myelin and posterior synaptic membranes. These receptors not only trigger and induce long‐term enhancement (LTP) in the hippocampus but also prevent myelin necrosis after white matter damage (Bliss, Collingridge, & Morris, 2014; Doyle et al., 2018; Lundgaard et al., 2013; Paoletti, Bellone, & Zhou, 2013). NMDARs are both ligand and voltage‐gated ion channels. They are heteropolymeric channels composed of NR1 and NR2 subunits that have high permeability to Ca^2+^. NR2 is a regulatory subunit, and NR2B is mainly distributed in the hippocampus of adult rats (Matta, Ashby, Sanz‐Clemente, Roche, & Isaac, 2011; Wenzel et al., 1995). There has been speculation that NR2B contributed most to the increase of NMDA‐evoked currents (Bjarnadottir et al., 2007; Dong et al., 2013; Kwag & Paulsen, 2012). The activation of NMDAR and Ca^2+^ influx plays a crucial role in the formation and survival of early neurons and the migration of nerve cells and synaptic formation during brain development (Hardingham, 2009). Under physiological conditions, NMDAR has a neuroprotective effect and can reduce apoptosis and neuroexcitatory toxicity. Normally activated NMDAR can allow passage of Ca^2+^ and mediate a physiological neuroprotective effect. When excitatory amino acids are over‐released and a large number of ion channels are activated, an NMDAR subtype will lead to the continuous increase of intracellular Ca^2+^ level, thus causing cell damage, which is called the toxic effect of excitatory amino acids (Dohare et al., 2014). In this study, both the expression level and phosphorylation level of NMDAR2B were significantly decreased in the hippocampus of SAD model group, which should be closely related to the decreased memory retention ability of the mice; this finding confirmed that LTP inhibition caused by the decreased NMDAR2B would damage memory function.

Metabotropic glutamate receptors (mGluRs) belong to the G protein‐coupled receptor family, which affects the second messenger system involved in regulating nerve excitation, synaptic formation, and nerve degeneration in many cells. mGluRs have a variety of subtypes, mainly distributed in the nervous system; among the mGluRs, mGluR5 is located in the excitatory posterior membrane and mediates the synaptic excitatory transmission caused by changes in glutamate content, thereby affecting synaptic plasticity and learning ability (Bruno et al., 2017; Pillai & Tipre, 2016). mGluR5 played an important role in a study of Aβ‐induced synaptic plasticity disorders (Yongan, Zheng, & Haiqiang, 2016).

Both mGluR5 and NMDAR are located in the posterior membrane of the synapse; they are directly connected through a series of skeletal proteins, such as Homer1b and Shank3, and their functions interact with each other (Pisani et al., 2001). They interact functionally: mGluR5 promotes NMDAR function by acting on Mg^2+^, which mediates the blocking effect of NMDA‐gated ion channels on Mg^2+^; in turn, the activity of NMDAR can reduce desensitization of mGlu5, thereby increasing its activity. Types of LTD in synapses in the CA1 region of hippocampus include NMDAR‐ and mGluR5‐dependent LTD. Under low‐frequency electrical stimulation, mGluR5 plays a catalytic role in NMDA‐dependent LTD, and both NMDAR and mGluR5 were coactivated when glutamate spillover occurred. However, NMDA‐dependent LTD induced under a different specific stimulus condition appears to be independent of mGluR5 (Bingjie, Fangyi, & Wensheng, 2017; O'Riordan, Hu, & Rowan, 2018).

In this study, with the decrease in NMDAR2B expression in the model group, the expression of mGluR5 was also decreased, which confirmed their synchronization. After treatment with donepezil or high‐dose SZL oral liquid, the mGluR5 and NMDAR protein levels in the hippocampus were higher than those in the model group, which was consistent with the improvement of their memory retention ability in the step‐down test. This indicates that donepezil and SZL oral liquid can increase synaptic connections, strengthen synaptic signal transduction and save SAD memory damage by increasing the content of mGluR5 and NMDA.

Glycogen synthesis kinase 3β (GSK3β) is a ubiquitous intracellular serine/threonine protein kinase, which was originally found to be involved in insulin regulation of glucose metabolism and cell survival. In a study of Parkinson's disease, activation of GSK3β can increase the autophagy level of cells, thereby enhancing the cell's ability to degrade α‐synuclein, which is a family of proteins regulating the release of neurotransmitters, mainly expressed in the presynaptic membrane; the inhibition of GSK3β will have the opposite effect on α‐synuclein (Bingjie et al., 2017; O'Riordan et al., 2018). Since α‐synuclein not only maintains normal synaptic function but also plays an important role in the degenerative diseases of the nervous system, it is presumed that GSK3β plays a pivotal role in the maintenance of synaptic function and in neurodegenerative diseases. The activity of GSK3β is regulated by phosphorylation levels, and the activity of GSK3β is inhibited when it is phosphorylated at Ser9. Its activation can inhibit the survival of some neurons, while its inactivation can promote the survival of neurons and improve the stability of cell structure (Dell'Osso, Del Grande, Gesi, Carmassi, & Musetti, 2016; Patel, Doble, & Woodgett, 2004). Several pieces of evidence have indicated that the level of phosphorylated GSK3β (p‐GSK3β) increased after LTP was induced in the hippocampus, suggesting that LTP is associated with the inactivation of GSK3β (Hooper et al., 2007). LTP was also inhibited when overexpression of GSK3β was observed in cortical and hippocampal neurons of mice, a phenomenon that was rescued by administration of GSK3β inhibitors (Peineau et al., 2007). Overexpression of GSK3β was also associated with cognitive decline in another behavioral test (Hernandez et al., 2002). In this study, STZ bilateral ventricular injection can damage the insulin‐signaling pathway in the CNS. The levels of p‐GSK3β decreased and the levels of GSK3β increased in the model group. After drug treatment, the expression of GSK3β decreased in donepezil‐ and high‐dose‐SZL‐treated groups, while its phosphorylation level increased in these two groups. Combined with the improvement of memory ability in drug‐treated mice, we speculate that SZL and donepezil could both improve synaptic plasticity and promote neuronal cell survival by influencing GSK3β, thus potentially playing a role in the treatment of SAD.

## 6 CONCLUSION

Donepezil is primarily used in clinical applications as a cholinesterase inhibitor; donepezil also maintains neuroprotective effects by reducing the neurotoxicity of Aβ, reducing the neurotoxicity of glutamate, and inhibiting excitotoxic injury (Zhang & Gordon, 2018).

Shenzhiling oral liquid is derived from a traditional Chinese decoction, the Kai Xin preparation, and is approved by the China Food and Drug Administration (CFDA) (Z20120010) for the treatment of mild‐to‐moderate AD; SZL consists of the following ingredients: *Codonopsispilosula*, Cinnanomi Ramulus, *Paeonialactiflora*, radix glycyrrhizaepreparata, poria, ginger, *Polygala*, grassleaf sweetflag rhizome, and fossilized and common oyster shell. Currently, no studies have shown that SZL has toxic and side effects. A previous systematic review suggested that SZL has beneficial effects on AD (Wang et al., 2019). Through research, we found that high‐dose treatment with SZL can increase the number of hippocampal synapses, affecting the levels of synaptic plasticity‐related proteins NMDAR2B, mGlu5, and GSK3β; SZL has a similar or even better neuroprotective effect as the positive control drug, donepezil. SZL oral liquid provides a potential possibility for the treatment of mild‐to‐moderate SAD, which is the new target of future research.

## Data Availability

The data that support the findings of this study are available from the corresponding author upon reasonable request.
